# Jejunal Enterolith: A Rare Case of Small Bowel Obstruction

**DOI:** 10.7759/cureus.8427

**Published:** 2020-06-03

**Authors:** Chantal Patel, Ravivarma Balasubramaniam, Timothy Bullen

**Affiliations:** 1 Surgery, University Hospitals of North Midlands, Newcastle-under-Lyme, GBR; 2 Radiology, University Hospital of North Midlands, Newcastle-under-Lyme, GBR

**Keywords:** enterolith, small bowel obstruction, jejunal diverticulosis

## Abstract

Small bowel obstruction is a common operative finding following an acute surgical admission. However, small bowel obstruction due to an enterolith is a rarer finding. Enteroliths are formed in conditions contributing to hypomotility and stasis within the gastrointestinal tract. These include Crohn’s disease, strictures, and intestinal diverticulae. We present a case of small bowel obstruction due to an enterolith in an 89-year-old female. In our case, CT identified an inflamed jejunal diverticulum pre-operatively.

## Introduction

Small bowel obstruction is a common finding following an acute surgical admission. The common causes of small bowel obstruction include adhesions, malignancy, and incarceration within abdominal wall hernias [1]. Here, we present an unusual case of small bowel obstruction due to an enterolith.

## Case presentation

An 89-year-old female presented to the surgical admissions unit with a two-day history of abdominal pain. This was associated with nausea and vomiting, with no bowel movements for the previous five days. The patient had also noted weight loss prior to admission. Her past medical history included diverticular disease, hypertension, and hyperthyroidism. There was no history of previous abdominal surgery. On examination, the abdomen was soft with lower abdominal distention and palpable bowel loops. 

**Figure 1:**
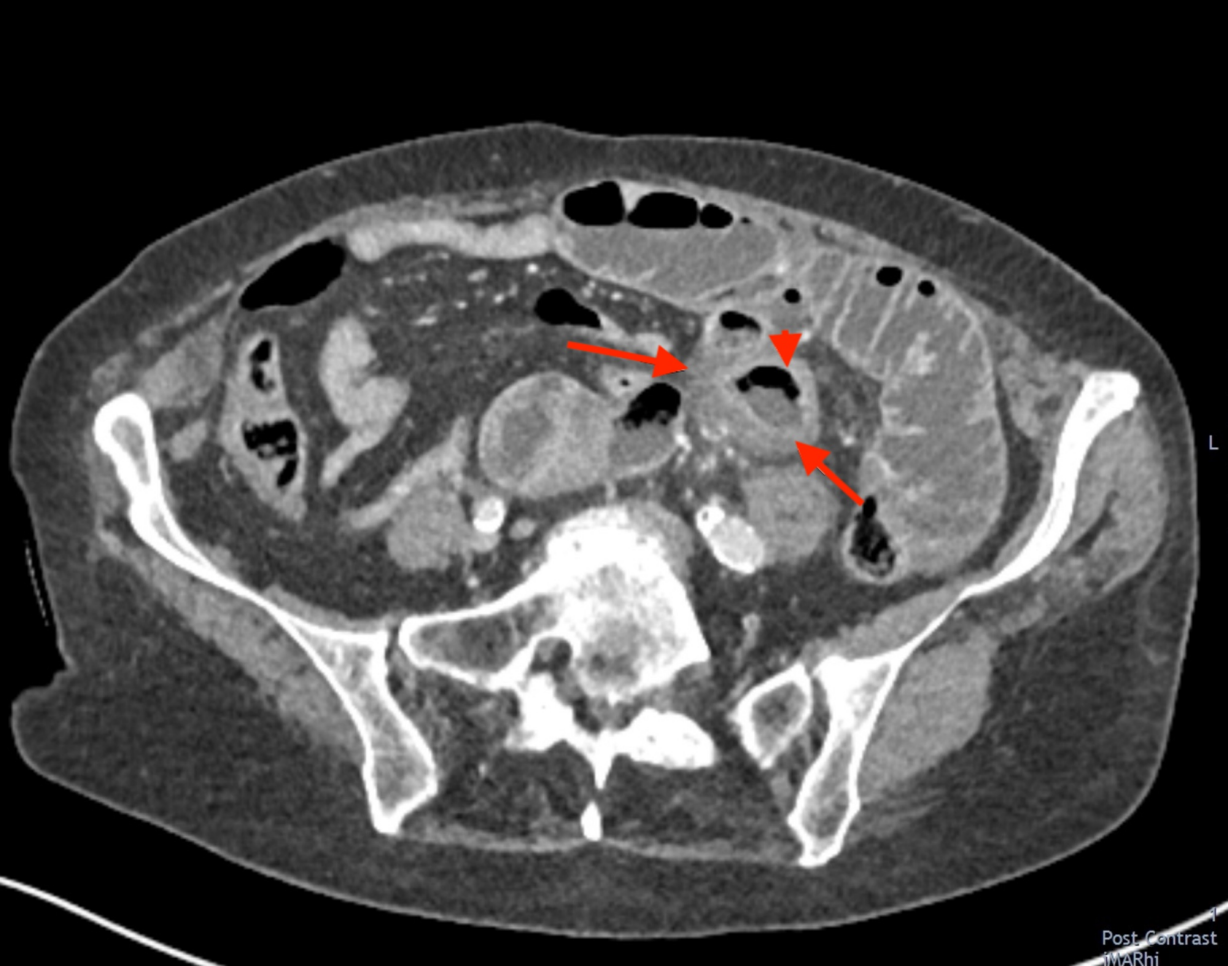
Axial CT image of an inflammed jejunal diverticulum (arrowhead). This is likely to be the originating site of the obstructing enterolith. The arrows highlight features of acute inflammation with wall thickening and surrounding mesenteric inflammatory stranding.

**Figure 2:**
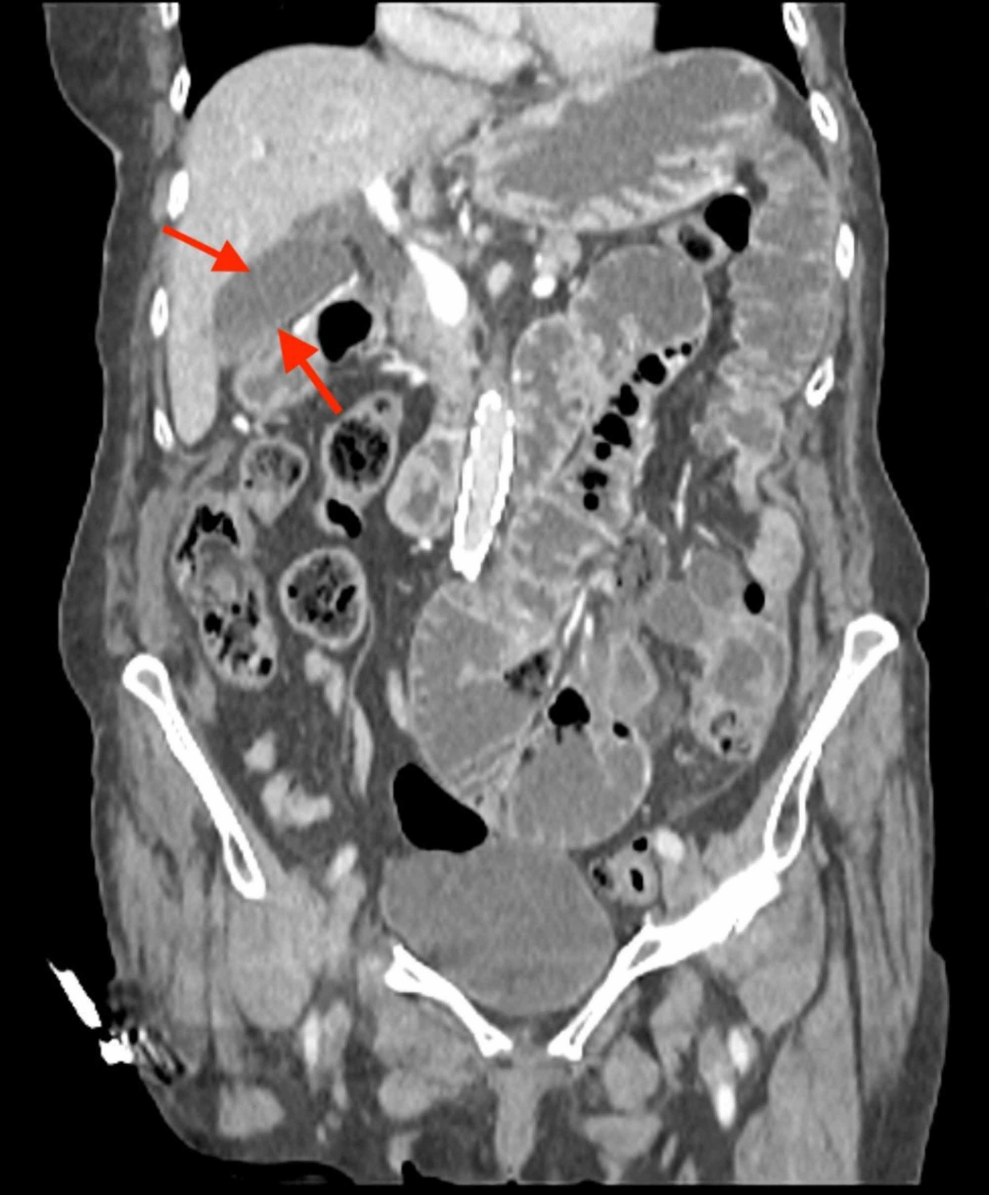
Dilated fluid-filled small bowel loops in keeping with a high-grade mechanical small bowel obstruction. Note the normal appearances of the gallbladder (arrows) and biliary tree that do not contain any gas (pneumobilia), which helps exclude the main differential of gallstone ileus.

**Figure 3:**
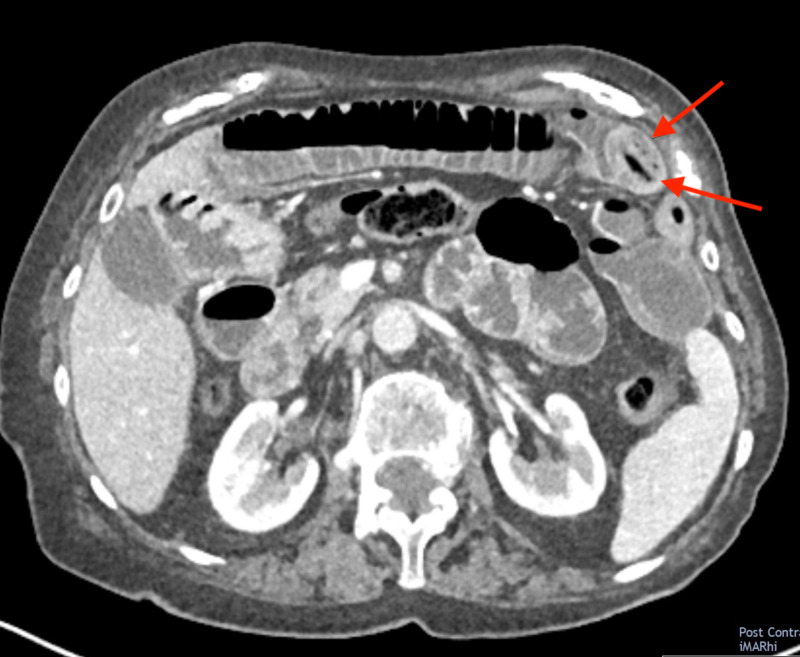
Axial CT image showing an obstructing enterolith (arrows) that has likely migrated from its original location in the inflamed jejunal diverticulum (see Figure 1) and now appears lodged in a loop of mid-small bowel.

**Figure 4:**
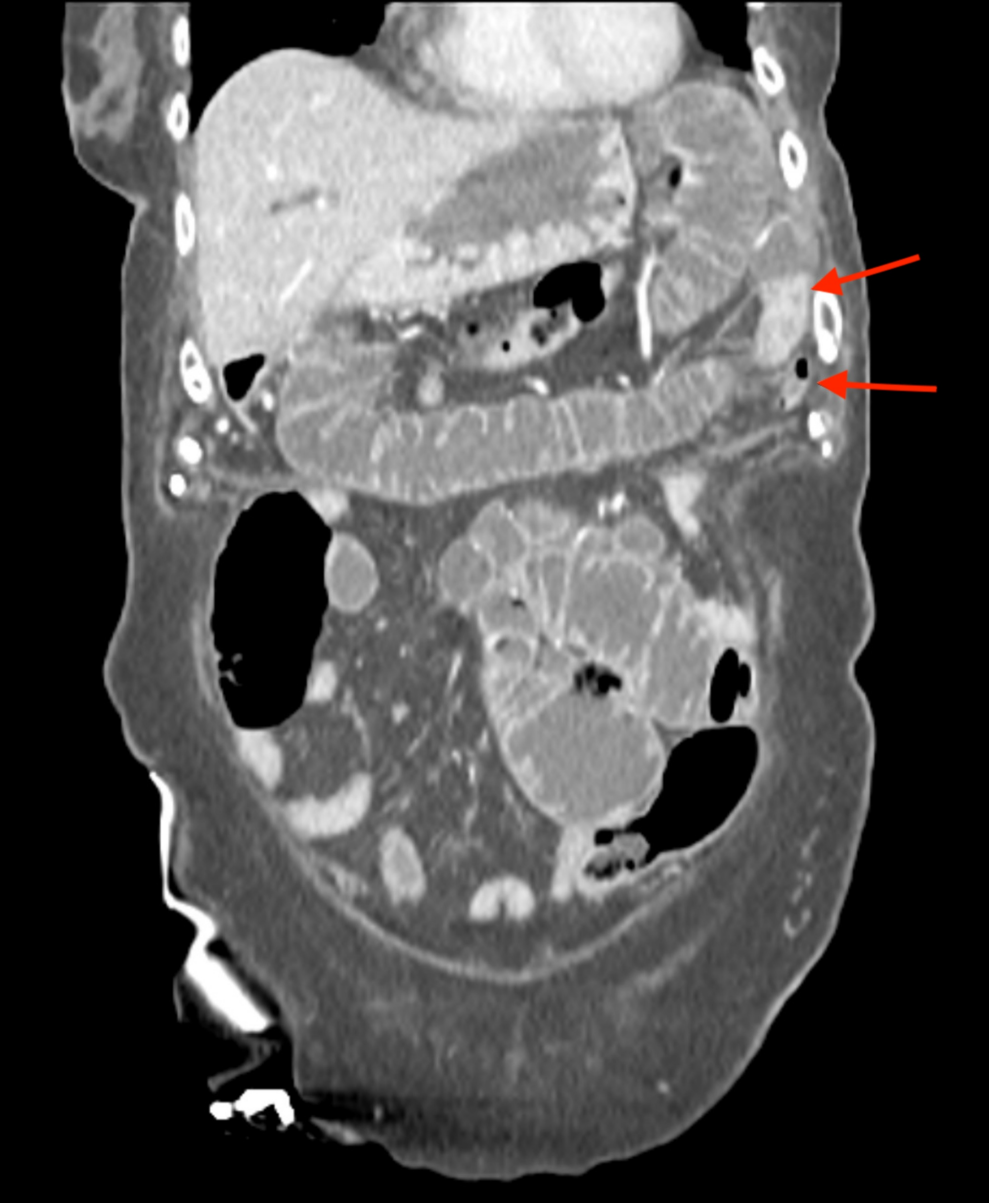
Coronal CT image showing the enterolith causing a high-grade mechanical bowel obstruction. This image demonstrates collapsed small bowel distal to the transition point (arrows).

Blood results revealed a lactate of 1.7 mmol/L and a C-reactive protein of 89.2 mg/L. All other serum blood results were in the normal range. CT of the abdomen and pelvis revealed contiguous fluid-filled dilated small bowel loops in keeping with high-grade mechanical bowel obstruction (Figures 1-4). After resuscitation, she was taken to theatre, and intra-operatively an enterolith was identified as the cause of the small bowel obstruction. 

**Figure 5:**
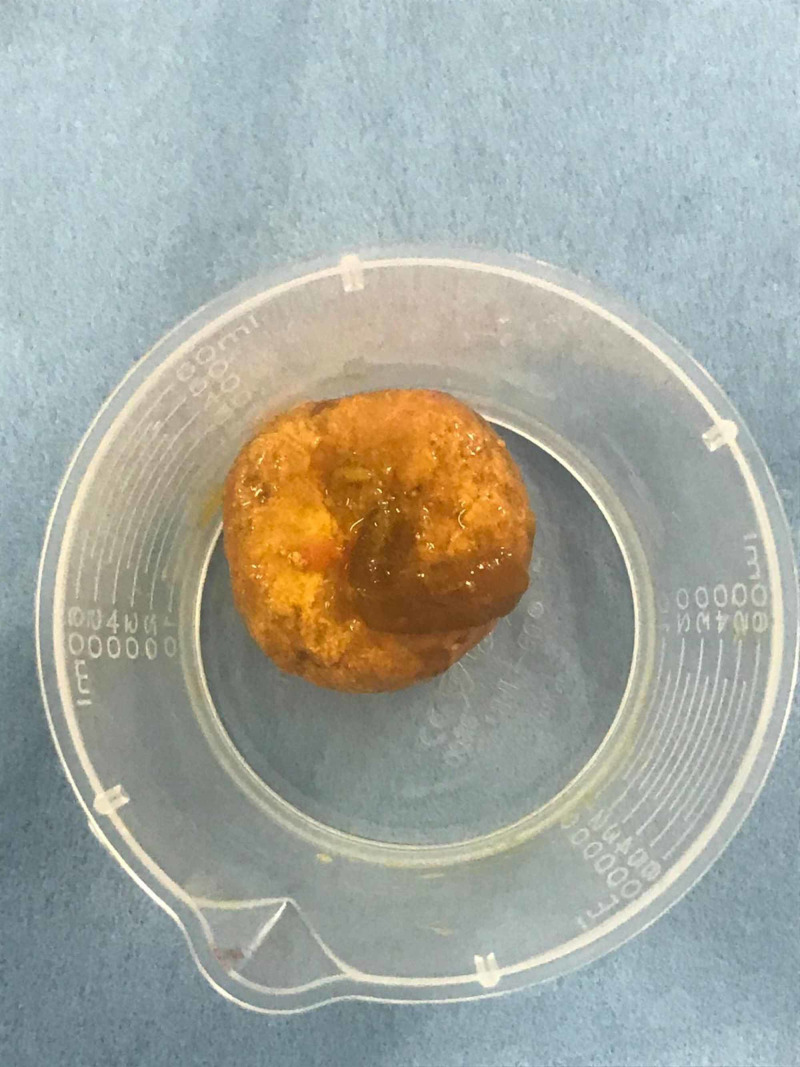
The enterolith was approximately 4 cm in diameter. It was identified and extracted intraoperatively.

The enterolith was milked proximally and removed via a transverse enterotomy (Figure 5). The gallbladder was thin walled with no palpable gallstones or fistulae. However, several jejunal diverticula were noted, one of which appeared to be inflamed. Following surgery, the patient returned to the ward for analgesia and supportive care. She recovered well and was discharged seven days following admission.

## Discussion

The causes of small bowel obstruction can be classified as extrinsic, intrinsic, or intraluminal (Table 1). Intraluminal causes of small bowel obstruction are rare, but include gallstones, worm bolus, bezoars, and enteroliths [2]. 

**Table 1:** Causes of small bowel obstruction A table shows the commonness of causes of small bowel obstruction

	Common	Less common	Rare
Extrinsic	Adhesions, hernias	Volvulus	Neoplasm
Intrinsic	Intussusception	Neoplasm, Crohn’s disease, stricture (post-radiation, ischaemic)	Diverticulitis
Intraluminal	-	Gallstone ileus, meconium Ileus, foreign body	Bezoars, enterolith, parasite

Enteroliths are mineral stones found within the gastrointestinal tract and can be classified as either primary or secondary enteroliths [3]. Primary enteroliths are formed within the gastrointestinal tract and secondary enteroliths are formed extrinsically, e.g. gallstones or kidney stones that have subsequently migrated into the intestines [1]. Enteroliths may also be classified as either true or false depending on composition. True enteroliths are formed of chyme and can consist of bile acids, calcium phosphate, and calcium oxalate [4]. Constituent minerals reflect the site and pH of formation within the tract [3]. False enteroliths include varnish stones, bezoars, chalk, and faecoliths [1].

Enteroliths are formed in conditions contributing to hypomotility and stasis within the gastrointestinal tract [5]. These include Crohn’s disease, traumatic or post-surgical strictures, anastomotic sites, and intestinal diverticulae [1,6].

In our case, several jejunal diverticula were noted intra-operatively and have contributed to the formation of the enterolith. Diverticula are found in the small intestine at sites of vessel insertion and are proposed to be caused by increased intraluminal pressure, bowel dyskinesia, or altered peristalsis [7]. They are commonly found in the duodenum, then the jejunum and most uncommonly in the ileum [8]. Concurrent colonic diverticular disease is often present [9]. The majority of patients with small intestinal diverticula are asymptomatic, but may present with abdominal pain, flatulence or malabsorption [9]. Complications include small bowel obstruction, haemorrhage, volvulus, intussusception, and diverticulitis, and may warrant surgical intervention [7].

Abdominal plain films undertaken in patients with a suspected small bowel obstruction may not highlight obstructing enteroliths as one-third of the stones are radio-opaque [10]. The utilisation of CT may increase the likelihood of diagnosing an obstructing enterolith; however, this was not immediately apparent in our case [1]. The use of ultrasound imaging in the diagnosis of small bowel obstruction due to enteroliths has been described in the literature [7]. Furthermore, imaging methods facilitate surrounding organs to be assessed for contributing secondary enteroliths, e.g. pneumobilia within the biliary tree in cases of gallstone ileus [5].

Small enteroliths less than 2 cm in size may be passed spontaneously with conservative management. The use of endoscopic retrieval or lithotripsy for duodenal enteroliths is also described. Surgical intervention is required for larger enteroliths. In some cases, the enterolith may be manually fragmented and allowed to pass via the large intestine. Where this is not possible, enterotomy and delivery of the enterolith is required [1]. Surgical intervention also facilitates the assessment of underlying pathology within the gastrointestinal tract and allows resection if necessary.

## Conclusions

We present a rare case of small bowel obstruction due to an enterolith. Furthermore, our case highlights jejunal diverticulosis as a contributor to enterolith formation. Enterolith is a rare but important cause of small bowel obstruction. Imaging will aid diagnosis only if the enterolith is considered as a cause and this may be easily overlooked. Surgical intervention is required in the majority of cases.
